# An Echo from the Past

**Published:** 1912-07

**Authors:** 


### An Echo From the Past.

One of the curiosities of medico-political news that has just come over the wires is that the Governor of South Carolina has vetoed a bill passed by the Legislature of that State for the medical inspection of school children. The name of this archaic individual is Blease, and he is a lawyer by profession. The grounds of the veto are that the proposed legislation is an outrageous violation of the rights of the individual. For a number of years past we have supposed that the doctrine of *laissez faire* has been regarded as obsolete by all people pretending to education and observant of the present evolution of society. But the lawyer, with his face turned perennially into the past, looking for precedents, can not see that the impudent interrogative answer of Cain when questioned by the Almighty concerning his brother, will not be permitted in the twentieth century any more than it was in the year one. The answer to the question, "Am I my brother's keeper?" is affirmative now as it was then. The South Carolina Teachers' Association, the South Carolina Medical Association, the South Carolina Federation of Women's Clubs and the General Assembly of that State are, fortunately for the people of the commonwealth, sufficiently educated and sufficiently alive to their duties to their brethren to have heartily endorsed this most necessary measure. To their credit be it said that the most important daily newspapers of the State also support the bill, and the *Columbia State* very pertinently asks:

"If the mothers, the teachers, and the doctors of South Carolina are not excellent judges of the legislation necessary for the protection of the health of children in South Carolina, to whose judgment will the people defer? Whose opinion, in opposition to the women, the doctors and the teachers of the State, who have given to this subject diligent study and investigation, shall be set up? Who is prepared to say that these thousands of experts are, after all, stupid cranks without understanding of the ways and means by which the health and happiness of the little children in the common schools may best be conserved?

"If it be held by the majority of the people of South Carolina that the recommendations of the teachers and doctors of the State should not be heeded in this matter, common sense and common consistency require that the teachers and doctors now ministering to the children and the families of South Carolina be dismissed and that others be employed in their stead."

Governor Blease's position is absolutely untenable and has never been seriously assumed in any other State in the Union by any supposedly sane and responsible citizen or body of citizens. For the credit of the State that he serves so ill in the executive chair he appears to stand alone in this matter and to be absolutely without support. In the light of this event and as long as we accord the foolish old monarchical right of veto to our governors, perhaps it would be well to abolish the elective character of that office, put them all on a civil service basis, and compel them to pass rigid examinations on constitutional law, history, commerce, mining, farming, transportation, sanitation and current events—and especially on sanitation and current events.—*Lancet-Clinic.*

